# Lumbar Colotomy for Obstruction of Rectum

**Published:** 1874-08

**Authors:** John H. Packard


LUMBAR COLOTOMY FOR OBSTRUCTION OF THE RECTUM.—*By John H. Packard, M.D.*—* * * The oblique incision seems to me to offer great advantages over either the vertical or the transverse; it affords more room to work in than the former, especially where the space between the last rib and the crista ilii is limited; and it is more easily kept closed than the transverse—at least if, in the latter, any of the fibres of the quadratus lumborum muscle are divided.

The rule given by Allingham and others, to divide the deeper layers of tissue to the same extent as the outer, lest the operator find himself working at the bottom of a conical hole, is a very important one. It must be remembered that the operation is undertaken in order to afford a free exit to the contents of the bowel; and this will not be accomplished if the deeper structures are insufficiently divided. Nor is anything gained by limiting the extent of the deeper sections, since there is no additional control gained thereby over the evacuation of liquid feces, while the difficulty of getting rid of any solid masses is much increased. And, by parity of reasoning, the incision through the wall of the bowel should be of ample size—an inch at least; this makes the securing of the edges to those of the wound in the skin much easier, and does not add to the risk of protrusion of the bowel.

An adequate number of silver wire sutures should be used to insure the complete contact of the everted wall of the gut with the skin; and these sutures need not be disturbed for weeks, unless they give rise to distinct annoyance by the twisted ends irritating the tissues.

